# The Coexisting Neuromyelitis Optica Spectrum Disorder and Systemic Lupus Erythematosus: A Therapeutic Challenge

**DOI:** 10.31138/mjr.20230808.tc

**Published:** 2023-08-08

**Authors:** Abhishek Kumar, Anirban Gupta, Preeti Gupta, Vivek Vasdev, S Kartik

**Affiliations:** 1 Department of Rheumatology, Army Hospital Research and Referral, Delhi, India,; 2 Department of Neurology, Army Hospital Research and Referral, Delhi, India,; 3 Department of Radiology, Command Hospital, Alipore, Kolkata, India

**Keywords:** neuromyelitis optica, Devic’s disease, central nervous system systemic lupus erythematosus, transverse myelitis, anti-aquaporin-4 antibodies

## Abstract

Neuromyelitis Optica (NMO), or Devic’s disease, is an immune-mediated, usually relapsing, central nervous system (CNS) demyelination disorder associated with optic neuritis and transverse myelitis. It is characterised by the presence of longitudinally extensive transverse myelitis (LETM) and antibodies against water channel aquaporin-4 (AQP4-immunoglobulin G [IgG]). The term NMO spectrum disorder (NMOSD) includes patients with limited forms of NMO who are at risk of recurrence. Often patients with NMO or NMOSD have an associated systemic autoimmune disease, most commonly systemic lupus erythematosus (SLE) or Sjogren syndrome (SS) or a related profile of non-organ-specific autoantibodies. The intriguing aspect of coexisting NMOSD and SLE is whether they are independent diseases that can coexist with each other or the serological findings specific to both diseases in a patient is a non-specific finding of no prognostic or therapeutic concern. We have presented two cases of NMOSD coexisting with SLE and based upon the existing evidence in the literature we present that the two conditions are independent of each other, and, at times, it can throw a therapeutic challenge to any clinician.

## INTRODUCTION

Neuromyelitis Optica (NMO) or Devic’s disease is an inflammatory central nervous system (CNS) disorder char-acterised by the presence of three or more vertebral segments longitudinally extensive transverse myelitis (LETM) and serum antibodies against water channel aquaporin-4 (AQP4-immunoglobulin G [IgG]).^1,2^ The term NMO spectrum disorder (NMOSD) was introduced in 2007 for AQP-4IgG seropositive patients with limited forms of NMO who were at risk of recurrence. It also includes patients with coexisting autoimmune disorders.^3^ The presence of AQP-4IgG in patients with the first episode of LETM is a strong predictor of its relapse or occurrence of optic neuritis in more than 50% of patients within 12 months.^4^

Patients with NMO or NMOSD often have an associated autoimmune disease, most commonly systemic lupus erythematosus (SLE) or Sjogren’s syndrome (SS), or a related profile of non-organ-specific autoantibodies.^5,6^ Customarily, patients with transverse myelitis and non-organ-specific autoantibodies have been classified as disease-associated transverse myelitis, irrespective of the fact that the disease has clinically manifested or not.^7,8^

There is no consistent opinion whether NMOSD and SLE/SS are independent diseases that can coexist with each other, or the serological findings are non-specific and AQP-4IgG or non-organ-specific antibodies can be seen in either condition. However, this differentiation has significant implications for the management of the two groups of diseases and, at times can be a therapeutic challenge when they coexist and are active in the same patient simultaneously. Here we present two cases of NMOSD coexisting with SLE and we have discussed the clinical implications subsequently.

## CASE 1

**Figure 1.**
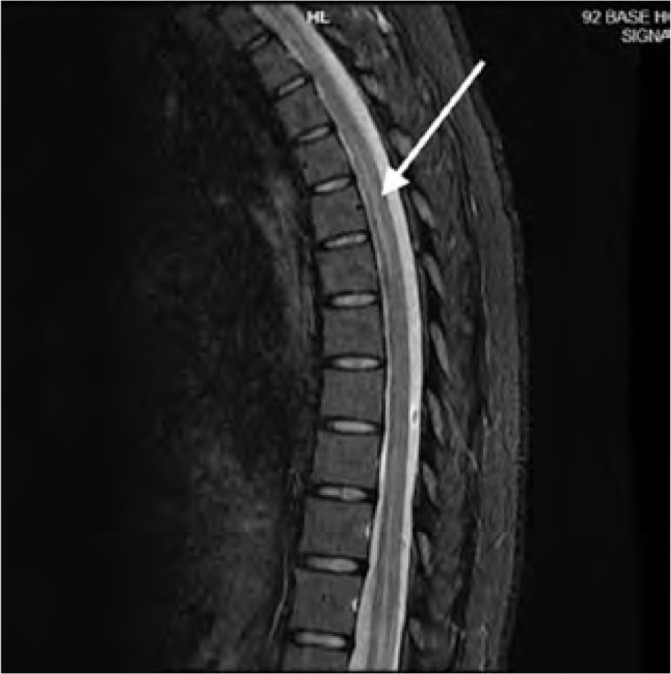
T2 hyperintensities of spinal cord at dorsal vertebral body levels D4-D6 (Case 1).

Our first case is a 27-year-old female patient who became symptomatic with pain in the lower abdomen and lower back on the same day followed by severe paraesthesia in the lower limbs. Four days later, she developed acute painful retention of urine and loss of bowel sensation, paraparesis, and loss of sensation to all modalities below the level of the umbilicus by the fifth day. She had a progressive course of illness and became bedridden with a complete inability to move her lower limbs. She was evaluated by a neurologist and a clinical diagnosis of transverse myelitis was made. Magnetic resonance imaging (MRI) of the spine showed long segment involvement of the spinal cord at dorsal vertebral levels 4 to 6 (D4-D6) with well-defined intra-substance T2 and short-tau inversion recovery (STIR) hyperintensities and seen to involve the central and both halves of the cord, sparing the dorsolateral part (**Figure 1**). There was no obvious cord swelling or atrophy or post-contrast enhancement. MRI of the brain was normal. Her blood counts and biochemical parameters were unremarkable. A detailed evaluation at our institute revealed a history of polyarthralgia with morning stiffness of one hour duration, hair loss for the last one year and malar rash for which rheumatology consultation was sought.

Further investigations at our hospital revealed normocytic normochromic anaemia (haemoglobin 10.7gm/dl) with normal leukocyte and platelet counts. Erythrocyte sedimentation rate (ESR) was raised (90 mm fall in the first hour), however, C-reactive protein (CRP) was normal (0.7 mg/L). She had proteinuria without any active sediment (24-hour urinary protein 1140 mg). Cerebrospinal fluid (CSF) analysis showed the presence of raised leukocytes (30 leucocytes/ml) with lymphocytes predominance and a mild increase in proteins (61mg/dl; a normal value less than 45 mg/dl). Other tests on CSF were normal (red blood cells-nil; no organisms in culture and staining; Sugar: 117 mg/dl [corresponding plasma glucose 137 mg/dl]; no oligoclonal bands).

Rheumatological evaluation of the patient showed positive anti-nuclear antibody (ANA) by indirect immunofluorescence (IIF) using Hep-2 cells and positive anti-double-stranded DNA antibody (dsDNA) positivity. Serum complements were normal. Lupus anticoagulants (LAC) were not present (negative LAC by diluted Russell’s viper venom time, negative anti-cardiolipin and anti-β2GP1 IgG and IgM antibodies). Anti-neutrophil cytoplasmic antibodies (ANCA) were negative. Other tests including serum angiotensin-converting enzymes (ACE), and serological markers for Human Immunodeficiency Virus and hepatotropic viruses were normal. However, kidney biopsy revealed International Society of Nephrology/Renal Pathology Society (ISN/RPS) class V lupus nephritis.^9^ The patient was diagnosed as a case of SLE (polyarthralgia, non-scarring alopecia, malar rash and proteinuria with positive ANA and anti-dsDNA antibodies fulfilling the Systemic Lupus International Collaborating Clinics [SLICC] criteria, 2012).^10^ However, due to the presence of LETM in MRI, there was a strong suspicion of NMOSD. Anti-AQP-4IgG antibody test by immunofluorescence was requested, and it turned out to be positive. Thus, the patient also fulfilled the international consensus diagnostic criteria for NMOSD, 2015.^11^ Involvement of the optic nerve was ruled out by a normal fundus, visual evoked potential, and MRI brain.

The patient was treated with methylprednisolone pulse followed by high dose oral prednisolone and B cell depletion therapy (rituximab). Significant improvement was achieved within two to three weeks with recovery of muscle strength to enable her to ambulate up to a hundred meters with minimal support. At five years of follow up patient had a complete recovery of neurological deficit and resolution of proteinuria.

## CASE 2

**Figure 2.**
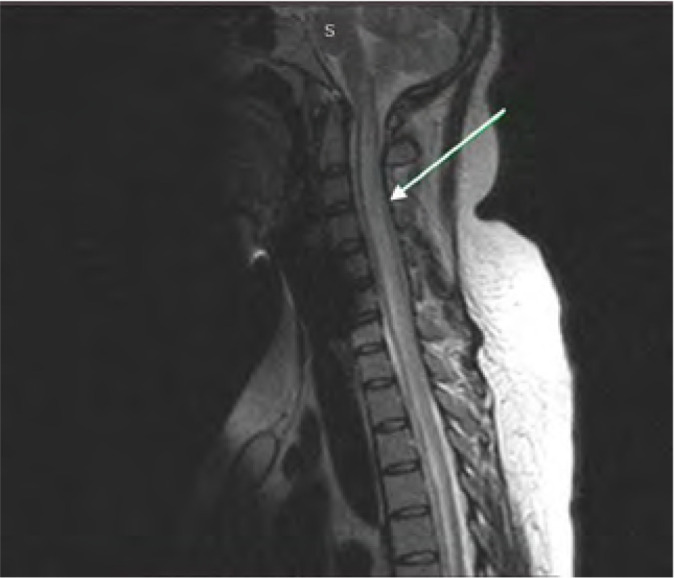
T2 hyperintensity with swelling of the spinal cord extending from the cervicomedullary region up to the axial level of the D12 vertebral body (Case 2).

Our second patient was a 34-year-old female. She was diagnosed to have SLE seven years ago when she presented with neuropsychiatric symptoms and discoid lupus erythematosus (DLE). She was doing well on azathioprine and hydroxychloroquine (HCQ) till 10 days before the presentation when she developed acute onset numbness over the left half of the body from mid-chest downward, weakness of left lower limb and acute retention of urine. Her symptoms gradually progressed to have numbness on the right half of her body as well and she developed weakness of neck muscles and trunk. On clinical examination, she had a loss of sensation to all modalities below T8 spinal level with grade 3/5 power in left lower limb and grade 0/5 power in right lower limb in all muscle groups. There was hypotonia and reflexes were diminished. There were no other manifestations to suggest SLE flare. MRI of the whole spine revealed long segment T2/STIR hyperintensity with swelling of the cord extending from the cervicomedullary region up to the axial level of the D12 vertebral body (**Figure 2**). It was predominantly seen in the central region of the spinal cord and involved most of the cross-sectional area. No post-contrast enhancement was seen. It was associated with swelling of the cord and mild prominence of the central canal. On further evaluation she exhibited the presence of anti- AQP-4IgG antibody by immunofluorescence. Anti-myelin oligodendrocyte glycoprotein (MOG) antibody was negative. CSF oligoclonal band was absent while CSF IgG was raised (82.1 mg/L, normal <34). No other abnormality was noted in CSF analysis. Her serum complements (C3 and C4) and CRP were normal and there was no proteinuria. A radiogram chest revealed no abnormality. Other haematological and biochemical parameters were normal.

She was diagnosed as a case of NMOSD and was given five days of pulse methylprednisolone (1000mg each). Due to a suboptimal response, it was followed by five cycles of plasmapheresis. For the maintenance phase, she was given rituximab. At the time of discharge, she could hold the neck of the pillow, could sit on the bed with little support and power in her hands had recovered. She could change sides on her own, left and right lower limb power was 4/5 and 2–3/5, respectively, and sensory loss was improving.

## DISCUSSION

Here we have presented two cases of SLE with LETM and AQP-4IgG positive status. Our Case 1differs from Case 2 in the manner that on the presentation itself, she fulfilled the classification criteria for both NMOSD and SLE (with lupus nephritis class V), while in Case 2, NMOSD was detected on the background of a long-standing SLE (without any evidence of active SLE).

Case 1 gave us several diagnostic and therapeutic challenges. Firstly, is the transverse myelitis a part of CNS manifestation of SLE or is it NMOSD coexisting with lupus nephritis? Does the treatment of transverse myelitis in SLE and NMOSD differ? If yes, should we treat transverse myelitis following the guidelines for the treatment of CNS lupus or NMOSD? If the existing evidence mandates treatment of NMOSD, in the prevailing circumstances, how should we manage the lupus nephritis class-V? We reviewed the literature to overcome this dilemma and we have discussed our opinion as below.

### Management of NMOSD

It includes treatment of acute episodes as well as prevention of recurrences. While prednisolone is the accepted recommendation for an acute episode, choices of a preventive agent are: 1^st^ line therapy - azathioprine or rituximab; second-line therapy - Mycophenolate mofetil (MMF) or Methotrexate or Mitoxantrone; third-line therapy - combination therapy (combination of steroids plus cyclosporine A or methotrexate or azathioprine) or Tocilizumab.^12^ Due to relatively higher rates of failure, cyclophosphamide is recommended only in case of failure or non-availability of other immunosuppressive therapy.^12^

### Management of SLE-associated severe

#### CNS manifestation

CNS manifestations of SLE consequent to thromboembolic pathology is treated with high dose prednisolone or pulse methylprednisolone and anticoagulation.^13^ Refractory cases can be subjected to immunosuppressive therapy in which intravenous cyclophosphamide has been found to be more effective than azathioprine or MMF.^13,14^ If vasculitis is possible pathogenesis for the CNS manifestation, high-dose prednisolone is recommended, followed by early institution of cytotoxic therapy for which, again, cyclophosphamide has been found to be more effective than other immunosuppressants..^13,14^ Once vasculitis has been controlled by cyclophosphamide, it can be substituted by another medication to maintain remission.^13,14^

#### Management of lupus nephritis

Mycophenolate mofetil and prednisolone remain the first-line treatment of ISN/RPS class V lupus nephritis, while calcineurin inhibitor alone or in combination with MMF is emerging as an alternative.^15^ Though the LUNAR trial failed to achieve the primary endpoint in cases of proliferative lupus nephritis, it has been reported to have a promising effect in several case reports and observational studies in membranous lupus nephritis.^16,17^

Thus, following the review of available literature, we concluded that if myelitis is secondary to NMOSD, the acute phase is treated by methylprednisolone and refractory cases should undergo rescue plasmapheresis. In the maintenance phase, azathioprine and rituximab become the first choice while cyclophosphamide is not recommended due to poor response. Conversely, if myelitis is considered to be the manifestation of SLE, cyclophosphamide becomes the preferred choice over rituximab and azathioprine.

#### The association of NMOSD and SLE

Sean J Pittock et al. in their study published in 2008 observed that anti- AQP-4IgG antibody is strongly associated with NMO, and SLE patients without a history of transverse myelitis lacked these antibodies.^18^ They also found that anti- AQP-4IgG antibody is not present in other conditions associated with severe myelopathy (like sarcoidosis, malignancies) or optic neuropathy. Moreover, the pathogenic role of anti- AQP-4IgG antibody in NMO is strongly supported by the presence of AQP-4 at the exact site of the NMO-typical vasculocentric deposition of immunoglobulins and products of complement activation in human NMO spinal cord lesions and selective loss of AQP4 immunostaining in all NMO lesions.^19,20^ Based on these pieces of evidence, they concluded that NMO and SLE are overlapping disorders that may coexist in some patients and the presence of non-organ-specific antibodies in patients of NMOSD who are positive for AQP-4IgG antibodies may reflect a more intense autoimmune response and such patients are at risk of developing other autoimmune disorders.^19.20^

Because of this evidence, we concluded that transverse myelitis in our patient was secondary to NMOSD and SLE was a co-existing condition and in any such condition wherein LETM with AQP-4IgG antibodies coexists with SLE, the transverse myelitis should be treated as part of NMOSD. A similar opinion has been promulgated by other reports as well.^21^ It has a significant therapeutic consequence which can be explained by a hypothetical situation. If our first case had ISN/RPS class IV LN, it would have mandated treatment for the same with either MMF or cyclophosphamide, which is not the drug of choice for NMOSD maintenance therapy. In such a condition, a combination of MMF with rituximab would have been a better choice. Our second case did not have any evidence of active lupus when she was diagnosed with NMOSD, so, we treated our patients with rituximab which is the first line long term therapy for NMOSD. The therapeutic import of the viewpoint mentioned above also holds relevance when newer drugs are being used to treat NMOSD. Eculizumab, a humanised monoclonal antibody against C5 complement was approved by United States Federal Drug Agency (USFDA) for maintenance therapy of NMOSD.^22^ Inebilizumab, another B cell depleting agent and satralizumab, an interleukin-6 (IL-6) inhibitor, have also been approved by USFDA for treating NMOSD in maintenance phase.^22^ Eculizumab has rarely been reported to be beneficial in special circumstances in patients with SLE.^23^ IL-6 inhibitors did not show any clinically meaningful response in trials.^24,25^ Although the B cell depletion with belimumab is an approved treatment for SLE, the evidence for other B cell depleting agents is insufficient or still emerging.^26^ Thus the therapeutic dilemma endowed with a coexistent NMOSD and SLE with its varied complications is likely to persist in near future.

## CONCLUSION

It can be emphasised that all cases of transverse myelitis or optic neuritis associated with the presence of any connective tissue disease or non-organ-specific antibodies, should be evaluated for the presence of anti- AQP-4IgG antibody. This antibody is highly specific for NMOSD and puts the patient at the risk of recurrence of myelitis or optic neuritis. Since recommendations for the treatment of NMOSD may differ from those of co-existing autoimmune condition, careful planning of treatment is required.
